# Expanding Horizons in Cardiac Transplant: Efficacy and Outcomes of Circulatory and Brain Death Donor Hearts in a Newly Implemented Cardiac Transplant Program with Limited Donor Accessibility and a Literature Review

**DOI:** 10.3390/jcm13174972

**Published:** 2024-08-23

**Authors:** Maria del Val Groba Marco, Miriam Cabrera Santana, Mario Galvan Ruiz, Miguel Fernandez de Sanmamed, Jose Luis Romero Lujan, Jesus Maria Gonzalez Martin, Luis Santana Ortega, María Vazquez Espinar, Francisco Portela Torron, Vicente Peña Morant, Eduardo Jose Caballero Dorta, Antonio Garcia Quintana

**Affiliations:** 1 Cardiology Department, Hospital Universitario de Gran Canaria Dr. Negrin, 35019 Las Palmas de Gran Canaria, Spain; mariogalvanr@hotmail.com (M.G.R.); mikefdsg@gmail.com (M.F.d.S.); ecabdor@gobiernodecanarias.org (E.J.C.D.); agarquil@gobiernodecanarias.org (A.G.Q.); 2 Departamento de Ciencias Medicas y Quirurgicas, Universidad de Las Palmas de Gran Canaria, 35016 Las Palmas de Gran Canaria, Spain; 3 Transplant Coordination Unit, Hospital Universitario de Gran Canaria Dr. Negrin, 35019 Las Palmas de Gran Canaria, Spain; micabsan@gmail.com (M.C.S.); vpenmor@gobiernodecanarias.org (V.P.M.); 4 Department of Critical Care, Hospital Universitario de Gran Canaria Dr. Negrin, 35019 Las Palmas de Gran Canaria, Spain; jromluj@gobiernodecanarias.org; 5 Research Unit, Hospital Universitario de Gran Canaria Dr. Negrin, 35019 Las Palmas de Gran Canaria, Spain; josu.estadistica@gmail.com; 6 Department of Anesthesiology, Hospital Universitario de Gran Canaria Dr. Negrin, 35019 Las Palmas de Gran Canaria, Spain; luisstna@yahoo.es; 7 Neurology Department, Hospital Universitario de Gran Canaria Dr. Negrin, 35019 Las Palmas de Gran Canaria, Spain; mvazespg@gmail.com; 8 Department of Cardiac Surgery, Hospital Universitario de Gran Canaria Dr. Negrin, 35019 Las Palmas de Gran Canaria, Spain; fportor@gobiernodecanarias.org

**Keywords:** cardiac transplant, cardiac surgery, heart failure, donation after circulatory death, donation after brain death, in situ perfusion, ex situ perfusion, thoraco-abdominal normothermic regional perfusion, donor pool

## Abstract

(1) **Background:** Cardiac donation after circulatory death (DCD) is an emerging paradigm in organ transplantation. However, this technique is recent and has only been implemented by highly experienced centers. This study compares the characteristics and outcomes of thoraco-abdominal normothermic regional perfusion (TANRP) and static cold-storage DCD and traditional donation after brain death (DBD) cardiac transplants (CT) in a newly stablished transplant program with restricted donor availability. (2) **Method:** We performed a retrospective, single-center study of all adult patients who underwent a CT between November 2019 and December 2023, with a follow-up conducted until August 2024. Data were retrieved from medical records. A review of the current literature on DCD CT was conducted to provide a broader context for our findings. The primary outcome was survival at 6 months after transplantation. (3) **Results:** During the study period, 76 adults (median age 56 years [IQR: 50–63 years]) underwent CT, and 12 (16%) were DCD donors. DCD donors had a similar age (46 vs. 47 years, *p* = 0.727), were mostly male (92%), and one patient had left ventricular dysfunction during the intraoperative DCD process. There were no significant differences in recipients’ characteristics. Survival was similar in the DCD group compared to DBD at 6 months (100 vs. 94%) and 12 months post-CT survival (92% vs. 94%), *p* = 0.82. There was no primary graft dysfunction in the DCD group (9% in DBD, *p* = 0.581). The median total hospital stay was longer in the DCD group (46 vs. 21 days, *p* = 0.021). An increase of 150% in transplantation activity due to DCD was estimated. (4) **Conclusions:** In a new CT program that utilized older donors and included recipients with similar illnesses and comorbidities, comparable outcomes between DCD and DBD hearts were observed. DCD was rapidly incorporated into the transplant activity, demonstrating an expedited learning curve and significantly increasing the availability of donor hearts.

## 1. Introduction

Cardiac transplant (CT) remains the most effective treatment for individuals suffering from advanced heart failure [1,2]. Traditionally, CT has mainly relied on organs from donors who were declared brain dead (DBD). However, the scarcity of suitable donor hearts poses a significant challenge and has led to the search for new strategies to increase the donor pool. One promising strategy is donation after circulatory death (DCD) [3,4], which is donation of the heart once it has been confirmed that circulatory function has permanently ceased.

Despite several differences in the process of organ recovery and storage, as well as ethical considerations, the early outcomes of DCD CT have been encouraging. Several DCD CT studies [5,6,7,8,9,10] and a recent randomized trial [11] report no difference in survival between DCD and DBD donor CT and show a significant potential to expand the donor pool, enabling more patients to receive these life-saving transplants.

However, DCD has many unanswered questions [12,13]. One of these issues is that this technique is recent, results have only been published by highly experienced centers, and the technique has an uncertain learning curve. Furthermore, with a better understanding of the technique, preservation strategies, tolerable ischemic times, and donor characteristics, previously underutilized DCD hearts may continue to be used, and CT programs should explore their limitations.

On the other hand, regions with restricted access to donors, for example CT programs in distant regions, encounter challenges in accessing donated organs given the limited potential for DBD and the ischemic time frontier. Therefore, it is crucial to explore and implement innovative strategies to expand the donor pool and lower the incidence of death or deterioration while on the waitlist.

In November 2019, a new CT program was initiated in the Canary Islands, Spain, which has a population of approximately two million people [14]. This was the initiation of the first CT in a European ultraperipheral region and faced two geographical challenges: a more than two-hour isolation from mainland Spain, and the multi-insularity of the seven islands that make up the Canary Islands. To increase the number of sourced donor hearts and contribute to a self-sufficient CT program, DCD with thoraco-abdominal normothermic regional perfusion (TANRP) and traditional static cold storage (SCS) was incorporated in an early phase of the program at our institution and, shortly thereafter, in all accredited donation hospitals in the Canary Islands.

Herein, we compare the characteristics and early clinical outcomes of TANRP-SCS DCD donor hearts with those of DBD in a newly established center. We also review the largest published clinical research with DCD hearts and update the current literature on this topic to give a broader perspective on our findings.

## 2. Method

### 2.1. Study Design

This retrospective, observational study included all adult patients (age > 16 years) who underwent CT at the Hospital Universitario de Gran Canaria Doctor Negrin, Spain, from November 2019 to December 2023, with a follow-up conducted until 6 August 2024. All CT were de novo, orthotopic procedures, performed using the bicaval technique.

Patients were classified into two groups: (1) heart from a circulatory death donor (DCD) and (2) heart from a brain death donor (DBD).

The required data were retrieved from our medical records and the Spanish Heart Transplant Registry, which included variables related to the recipient, donor, intervention, immunosuppression, and follow-up.

Organ allocation was conducted equally and transparently by the Spanish Organ Transplant Organization (ONT). The patients signed a general and a DCD-specific consent form. The study was approved by the Clinical Research Ethics Committee of the Hospital Universitario de Gran Canaria Dr. Negrin (Las Palmas, Spain) and was conducted in compliance with the Declaration of Helsinki.

### 2.2. Organ Procurement and Management

DBD hearts with SCS are considered standard care, remaining the standard practice in most transplant units.

DCD donated hearts are considered in patients who died following a planned withdrawal of life-sustaining treatment (WLST) (Maastricht category III) or in patients with an intentional ending of life-euthanasia (Maastricht category V).

The DCD program follows the protocol implemented in all Spanish DCD CT programs [15]. The donation process respects the wishes and values of patients and their relatives. Once support measures are withdrawn, a 5 min “no touch period” to confirm circulatory arrest is required by the Spanish law. Subsequently, a super-rapid sternotomy with clamping, cannulation, and drainage of the three supra-aortic trunks (SAT) or cephalic section of the SATs with blood recuperator systems (last modification of the protocol in March 2023) are carried out to guarantee the absence of cerebral reperfusion [16]. Artificial circulation using extracorporeal oxygenation circuits (ECMO) or extracorporeal circulation machines is initiated to perfuse the organs during recovery. Monitoring of the donor with the bispectral index and transcranial Doppler (TCD) is used to confirm the absence of cerebral perfusion. During machine perfusion, the donor heart is assessed by transesophageal echocardiogram, hemodynamic parameters, and in situ visualization. After a satisfactory heart function is recovered, TANRP is gradually weaned off, and the heart re-assessed in physiological conditions. The heart is then extracted and preserved using SCS.

In donors who are available in other secondary care and spoke hospitals, fluent communication among prehospital care providers is maintained, and an organized plan is developed for programmed donation. A mobile material and personal retrieval team is organized to support hospitals lacking this technology and knowledge. Local airways transport our multidisciplinary team, formed by a cardiac surgeon (ECMO cannulation and cardiac extraction), cardiologist (supports the validation of the heart using transesophageal echocardiogram), transplant coordinator (coordinates the procedure), critical care doctor and anesthesiologist (Swan Ganz monitoring and care assistance), neurologist (confirms absence of cerebral perfusion using TCD), and two nurses (transplant nurse coordinator and organ perfusion specialist), as well as the required material (including an ECMO system). Once the heart is validated, the organ is transported using SCS by the emergency public transport system to our hospital, where transplantation is performed.

### 2.3. Clinical Outcomes

The primary endpoint of the study was recipients’ survival at 6 months after transplantation. The secondary endpoints included the extracorporeal circulation time, length of intensive care unit (ICU) and total hospital stay, median time spent on a ventilator, severe primary graft dysfunction (PGD) defined as dependence on left or biventricular mechanical support, postoperative atrial fibrillation, acute renal failure, renal replacement therapy (RRT), and 30-day and 1-year survival.

### 2.4. Statistical Analysis

The median and quartiles were calculated to describe the quantitative variables. The Kolmogorov–Smirnov test was used to check the normality of the quantitative variables. Qualitative variables were described by absolute and relative frequencies. The non-parametric Mann–Whitney U-test was used to compare the distribution of numerical variables in the two cohorts because of the small sample size of one cohort. Fisher’s exact test was used to examine the association between qualitative variables. The Kaplan–Meier (KM) method was used to construct the survival curves, and the log-rank test was used to compare the survival curves. Statistical significance was set lower than 0.05. The statistical program used was R Core Team 2023, version 4.3.2.

## 3. Results

### 3.1. DCD and DBD Recipients, Donors, and Basal Characteristics of Procedure

**Figure 1:**
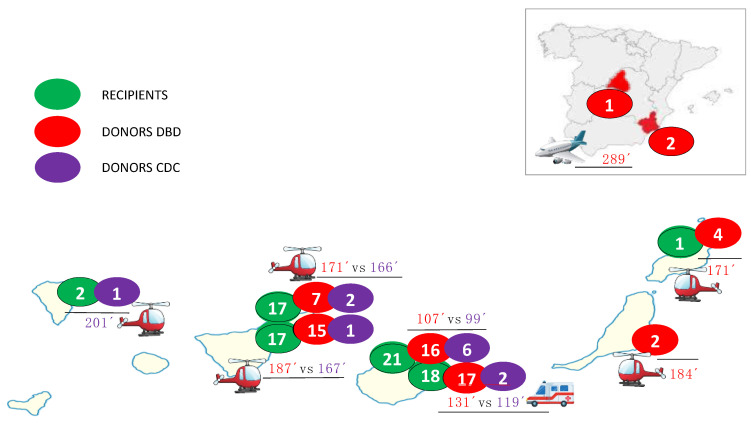
The origins of recipients and donation after brain death (DBD) and donation after circulatory death (DCD) donors; ischemia times.

From November 2019 to December 2023, 76 patients underwent de novo CT using the bicaval technique, which included 12 DCD donors (1 in 2021, 4 in 2022, and 7 in 2023) and 64 DBD donors. Nine DCD donors were Maastricht category III and three Maastricht category III. Figure 1 shows the origins of recipients, DBD and DCD donors, and mean ischemic times, and Table 1 shows the main recipients, donors, and procedure characteristics stratified by DBD or DCD.

Among the recipients, the median age was 57.5 years (IQR: 50.7–63 years), 16 (21%) were women, and 28 (37%) had ischemic heart disease. There were no significant differences in recipient characteristics between the two groups. Two DCD recipients were on temporary mechanical circulatory support at the time of transplantation (one patient was supported with a balloon pump and another on ECMO).

Compared to the DBD donors, the DCD donors had a lower weight (72 vs. 83 kg; *p* = 0.012) and body mass index (23 vs. 26; *p* = 0.01). However, there were no significant differences in age (46 vs. 47 years), sex (92 vs. 86% male), or comorbidities (diabetes mellitus, hypertension, and smoking). The donor left ventricular ejection fraction was similar in both groups (62.5 vs. 60%; *p* = 0.456). One DCD heart had dysfunction in the echocardiogram during in situ evaluation of the donor heart after TANRP was weaned off [17]. There were no significant differences in ischemia and extracorporeal circulation times (118 vs. 146 min, *p* = 0.106; and 96 vs. 103 min, *p* = 0.185).

The functional warm ischemic time ranged from 10 to 30 min (median 15 min, IQR 12.5–16 min). TANRP was weaned off or decreased to <1 L within 30 min. Among DCD, seven livers, eighteen kidneys, and four lungs were also recovered and transplanted. No heart allograft was discarded during organ procurement.

### 3.2. Survival and Secondary Outcomes

**Figure 2:**
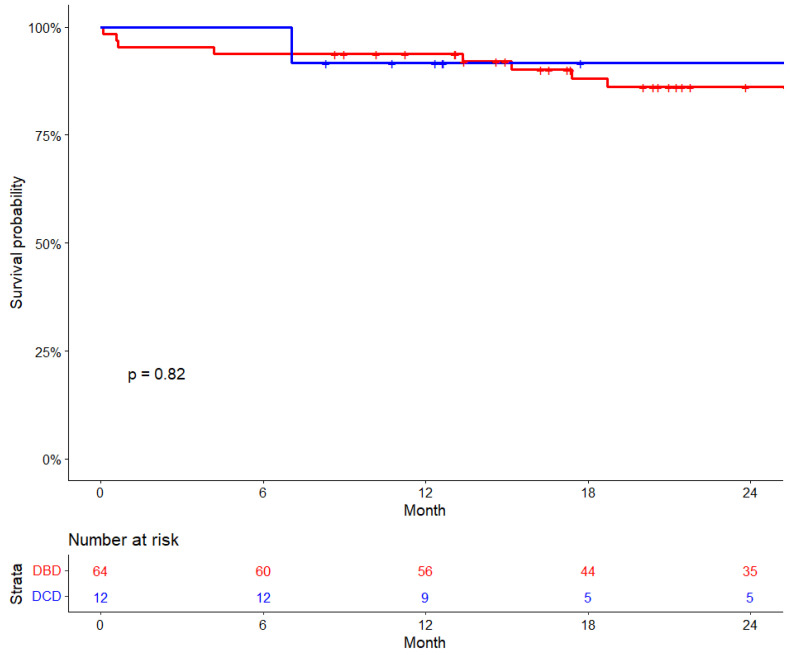
Cardiac transplant survival by donor type.

The median follow-up period for the overall cohort was 26 months (IQR: 14.9–38.9 months), and the KM estimates of 30-day survival were 96%, while 6-month survival was 95% and 1-year survival was 93%. For DCD recipients, the follow-up period was 15.2 months [IQR: 11.9–28.0 months], while for DBD recipients, it was 27.5 months [IQR: 16.5–42.2 months], *p* = 0.027. There were no significant differences in survival rates between DCD and DBD recipients. The 6-month survival for DCD was 100%, compared to 93.8% for DBD recipients. The 30-day survival was 100% for DCD and 95.3% for DBD, and the 1-year survival rate was 91.7% for DCD recipients compared to 93.8% of DBD recipients (*p* = 0.82) (Figure 2).

The median total hospital stay was longer in the DCD group (46 vs. 21 days, *p* = 0.021). There was no PGD in the DCD group (9% in the DBD, *p* = 0.581). The incidence of RRT in critical care unit showed a higher trend (58 vs. 28%, *p* = 0.052), although no DCD recipient was on RRT after ICU discharge (only one in the DBD group). There were no differences between groups in intubation time (9 vs. 8 h, *p* = 0.562). No DCD recipients required mechanical support after transplantation. Table 2 shows the main clinical post-CT outcomes for the DCD and DBD recipients.

### 3.3. Potential Impact of Adult DCD in the Canarian CT Program

It is difficult to estimate the potential impact of DCD in the global cohort because the first protocols had stricter donation criteria, and in 2023, the Spanish DCD program was stopped until the ethical aspects of the protocol technique were resolved. The increase in transplantation activity due to DCD was calculated as the number of DCD transplants divided by the number of DBD transplants, multiplied by 100. Therefore, if we only evaluate the last 6 months of the CT program (July–December 2023), six DCD and four DBD procedures were performed, which accounts for an estimated 150% increase in transplantation activity.

## 4. Discussion

To successfully implement a CT program, strategies must be devised to augment the donor pool and transplant activity to meet the growing demands for CT and reduce waiting list mortality [3,12]. Recent evidence suggests that using hearts recovered from DCD has become instrumental in replenishing donors following a decline in DBD potential and can boost CT activity by up to 30–48% [7,8]. Despite its promise, DCD CT programs are relatively recent, mostly performed in large-volume centers of excellence, and comes with an unknown learning curve, varying protocols with strict inclusion criteria, and ethical dilemmas influenced by different country legislations.

The first successful CT in 1967 used a donor heart resuscitated postmortem using cardiopulmonary bypass [13]. After the publication of the Harvard Commission Consensus Statement on brain death in 1968 and the recognition of neurological criteria in the Uniform Definition of Death Act (UDDA) in 1981 [18], the emergence of DBD techniques caused a shift away from the use of DCD in the field of CT. However, in recent years, the greater control of the DCD procedure, along with new models of extraction, preservation, and transport, has allowed for the resumption of DCD CT programs with promising results.

DCD is considered when WLST is determined, as continuing treatment will not allow the patient to survive or will not result in a functional outcome with an acceptable quality of life that the patient and the treating team regard as beneficial. The Maastricht classification (Table 3) distinguishes several categories of potential donors in different end-of-life situations: DCD occurring in an uncontrolled context (categories 1 and 2), controlled DCD (categories 3 and 5), or both (category 4) [19]. In category I, patients are declared “dead on arrival”. Category II involves cases where resuscitation attempts are unsuccessful, regardless of whether these attempts happen inside or outside the hospital. Category III represents the most common scenario, where both the treating physician and the family are “awaiting cardiac arrest” to officially declare the patient’s death. Category IV always involves a “cardiac arrest after brain death”. The unique situation under some countries´ law that permits euthanasia is detailed in category V, labeled “euthanasia”. This category includes patients who consent to medically assisted circulatory death.

**Figure 3:**
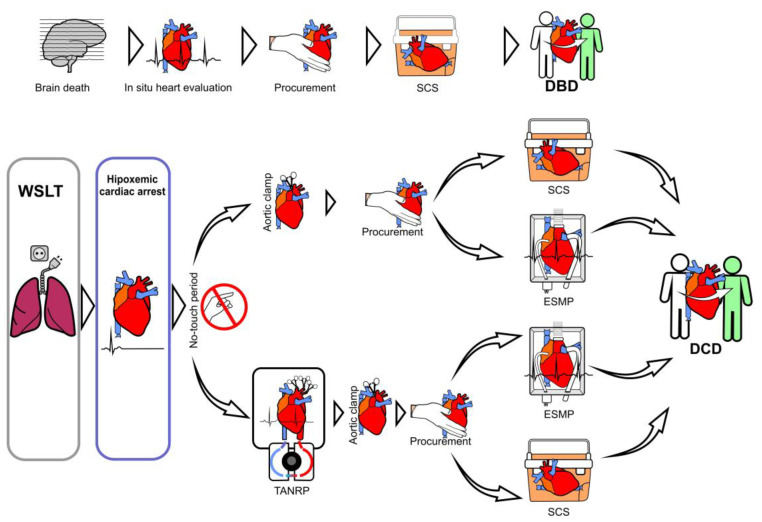
Types of donation: donation after brain death (DBD) and donation after circulatory death (DCD). Techniques of reperfusion in DCD hearts: reperfusion using direct procurement (DP) and static cold storage (SCS) or ex situ machine perfusion (ESMP); or in situ perfusion of the heart, known as thoraco-abdominal normothermic regional perfusion (TANRP), with ESMP or SCS.

Two main methods of reperfusion and four different techniques have emerged for the procurement and preservation of DCD hearts (Figure 3) [20]. The first two methods are performed by direct procurement (DP) after circulatory death with SCS and transplantation (in disuse) or ex situ perfusion outside the donor, using ex situ machine perfusion (ESMP), which allows the assessment of cardiac functionality before transplantation. The other two methods are performed inside the donor, using TANRP with an open cardiopulmonary bypass circuit or ECMO and subsequent ESMP or traditional SCS.

The largest published clinical studies with DCD hearts include studies from various countries (Table 4): one clinical trial [11], and recent retrospective studies [5,6,7,8,9,10] The techniques used for perfusion and procurement include DPP-ESMP [8,11], TANRP [5], and both techniques [6,7,9,10]. These studies report severe PGD rates that range from 5.7% to 34%, with a trend towards an increased PGD risk in the DCD group, although no significant difference was found. The DCD 30-day survival rates ranged between 96% and 100%, the 6-month survival between 93% and 94%, and the 1-year survival rates from 91% to 100%, without a significant difference between DCD and DBD recipients. The longest reported survival is 5 years, with rates of 84.3% and 88% [5,8].

As there are no standardized protocols on DCD CT, our institution´s protocol, following the Spanish model, is an in situ evaluation of the donor heart using TANRP and a multimodal assessment of cardiac functionality after death, which includes direct visualization, pulmonary artery catheter monitoring and echocardiography. This could hypothetically allow a better donor selection and evaluation of the donated heart [21].

There is limited research on TANRP. DPP-ESMP is the predominant heart procurement strategy in most retrospective studies, and there is no clinical trial on TANRP or prospective study analyzing TARNP versus DP-ESMP. However, different studies suggest that although no significant differences are observed between TARNP and DP-ESMP, TARNP has higher survival rates and lower PGD [6,7,9,10]. Also, TARNP is associated with increased rates of organ utilization, which can be attributed to the evaluation of the donated heart under physiological conditions. To date, no benefit has been demonstrated in terms of heart preservation with ESMP or SCS after TANRP [21].

TANRP is considered a cost-saving technique. For ex situ perfusion of DCD hearts, the TransMedics Organ Care System (OCS™) (Andover, MA, USA) is the only FDA-approved system and costs more than EUR 400,000, with an additional EUR 105,000 per perfusion procedure. In contrast, each CT performed with TARNP is estimated to cost less than EUR 6000 [22]. The San Diego program has calculated that the cost per transplant is 50 times lower with TANRP versus DP-ESMP [22].

It is important to emphasize that one of the main reasons for the limited generalization of DCD programs is the ethical dilemmas posed by DCD, especially when the heart is removed using TANRP techniques [12]. In this regard, more clinical studies are needed to standardize the TANRP protocols, continue ethical debates, and obtain legal support to achieve global medical and society acceptance, facilitating the consolidation of DCD programs [23].

The low total recipient and donor volume and the recent initiation of the CT program with unique geographical characteristics provide additional valuable insights into the feasibility, outcomes, and potential of DCD CT in new programs and in populations with restricted access to donors.

Concerns have been raised regarding inequality of a candidate´s probability of transplantation in smaller CT programs and the feasibility of DCD in settings with significant barriers to transplantation and low-volume centers [21,23]. It is well known that transplant programs undergo a learning curve, adapting and refining their processes over time to enhance their efficiency and outcomes. Messer et al. [7] report that three transplant centers with low experience of DCD transplantation had a 100% ECMO rate post-transplant, which could explain the significant post-transplant ECMO rate of 40% observed in their study. Over time, other studies have demonstrated that with increasing surgical experience in DCD recovery, PGD rates decrease [5].

This is the first study in the DCD era that reports the results of a newly initiated CT program with a medium of approximately 19 CT per year. After only 76 CT performed since its start in November 2019, DCD using TANRP and SCS was incorporated early, increasing the donor pool, with comparable results to previous larger-volume centers and no significant difference in survival or severe PGD compared to DBD recipients. Furthermore, the study adds evidence to the limited published data and experience with TANRP-SCS CT.

On the other hand, the shortage of donor hearts or restricted access to donations in certain regions necessitates the exploration of strategies to expand the donor pool and bridge the supply–demand gap, increasing the probability of transplantation. Our self-sufficient program achieves success with DCD hearts with certain expanded criteria compared to the results published to date in the literature, pushing the limits of this procedure.

Donor age is a relevant characteristic of our results that must be highlighted. In previous published clinical research with DCD hearts, donor ages ranged from 26 to 35 years and donors were significantly younger in the DCD group compared with those in the DBD group [5,6,7,8,9,10] (Table 4). However, our study included older donors, with a median age of 55 years, and 75% of recipients were older than 40 years. Furthermore, there were no significant differences between groups in terms of age: the median age for DCD was 55 years (IQR 32–62), compared to 58 years (IQR 50–63) of DBD donors (*p*-value = 0.792). This is especially important as expanding the donor age criteria could also augment the donor pool.

Similarly to what occurs with donors, previous studies report that DCD recipients had fewer comorbidities and were considered less ill prior to transplant compared to DBD recipients [5,9]. However, in our study, no significant differences were observed in the basal characteristics of DCD and DBD recipients, with a high proportion in DCD recipients of chronic kidney disease (83%), intravenous inotropes before CT (33%), and pretransplant circulatory support (two patients). Although it was initially thought to start with less acutely ill patients, the shortage and limited access to donors made DCD possible in these patients.

Despite the Canary Islands being a multi-island autonomous community, the established coordination was extremely precise, and ischemia times were very short in both groups (DCD 118 vs. DBD 146 min, *p* = 0.106). Figure 1 shows the mean ischemia times for each hospital procurement and donation type, without significant differences, even though 33% of DCD donors were from another island (43% of donor in DBD group from another island and 5% from mainland Spain), with the longest ischemia time being 201 min (a donor on the island of La Palma, 250 km away from the transplant center by helicopter). No DCD donor was retrieved from mainland Spain (three in the DBD group, with a maximum ischemia time of 305 min).

In our study, a higher trend of RRT rates during the immediate post-transplant ICU stay could be observed. This result is similar to other studies [6,7,10]. Although future investigation is needed to elucidate the etiology of this renal failure, possible explanations may be related to the higher rates of transition right heart dysfunction [16] and ECMO use [15]. In our cohort, a greater proportion of DCD recipients had chronic renal failure (83.3% vs. 64.1%, *p* = 0.317) and were receiving intravenous inotrope medication at the time of their transplant (33.3% vs. 28.1%, *p* = 0.72), which could also predispose them to RRT. This may also explain the longer total hospital stay observed in our DCD group (46 vs. 21 days, *p* = 0.021). However, previous studies report no significant differences in ICU or hospital stays^,^ [6,9,10].

Our study had some limitations. Firstly, it was subject to the inherent limitations of an observational and retrospective study design. The limited number of patients analyzed may have prevented us from demonstrating that some clinically relevant results were statistically significant, and a larger number of patients is required to confirm these findings. Extrapolation to other regions should be made with caution due to the specific particularities of our program, and we should await longer-term results. Further studies and clinical trials are needed to establish the optimal DCD heart recovery and preservation techniques. Future research should focus on comparing TANRP and DP-ESMP methods, evaluate long-term outcomes and costs, resolve ethical controversies, and harmonize regional practices with standardized protocols.

## 5. Conclusions

Comparable outcomes between DCD and DBD hearts are observed within a new CT program that utilized older donors and included recipients with similar illnesses and comorbidities in both groups. These findings support the feasibility and effectiveness of DCD, which was swiftly integrated into our transplant activity. The expedited learning curve and significant potential of DCD transplantation underscore its promise in addressing the critical donor heart shortage.

## Figures and Tables

**Table 1:** Baseline cardiac transplant characteristics based on DCD or DBD donor status.

Characteristics	DBD n = 64	DCD n = 12	*p*-Value
	RECIPIENTS		
Age, y	58 (50–63)	55 (52.75–61.75)	0.792
Male sex, n (%)	51 (80)	9 (75)	0.708
BMI	25.23 (22.08–27.21)	27.48 (24.18–30.17)	0.176
Ischemic etiology, n (%)	22 (34.4)	6 (50)	0.341
Insulin-dependent diabetes, n (%)	14 (21.9)	4 (33.3)	0.463
Moderate–severe COPD, n (%)	5 (7.8)	1 (8.3)	1
Chronic kidney disease, n (%)	41 (64.1)	10 (83.3)	0.317
Previous cardiac surgery, n (%)	10 (15.6)	1 (8.3)	1
PVR, UW	1.83 (1.4–2.44)	1.91 (1.36–2.29)	0.74
Creatinine, mg/dL	1.3 (1.02–1.63)	1.37 (0.88–1.84)	0.784
Bilirubin > 2 mg/dL	8 (12.7)	0	0.341
Urgent Transplant, n (%)	5 (7.3)	1 (8.3)	1
Intravenous inotropes before CT, n (%)	18 (28,12)	4 (33.33)	0.72
Time on waitlist (days)	40.5 (9.75–95.5)	11 (4.75–49.5)	0.127
Pretransplant mechanical ventilation (days)	5 (7.8)	1 (8.3)	1
Pretransplant circulatory support, n (%)	13 (20.3)	2 (16.7)	1
-None	48 (78.7)	9 (81.8)	
-Balloon pump	7 (11.5)	1 (9.1)	
-ECMO	4 (6.6)	1 (9.1)	
-Ventricular support	2 (3.3)	0	
	DONORS AND PROCEDURE		
Age, y	47 (35.75–57.25)	46 (42–48.25)	0.727
Male sex, n (%)	55 (85.9)	11 (91.7)	1
Weight, kg	83.5 (72.75–90)	72 (66.5–76.5)	0.012
BMI	26.18 (23.75–29.34)	23.4 (20.48–24.72)	0.01
Hypertension, n (%)	14 (22.2)	3 (25)	1
Diabetes Mellitus, n (%)	3 (4.8)	0	1
Current smoker, n (%)	26 (41.3)	3 (25)	0.349
Size mismatch	18.91 (2.76–41.12)	9.1 (−19.45–23.84)	0.2
Sex mismatch (Female donor–male recipient), n (%)	4 (6.2)	0	1
Donor left ventricular ejection fraction, %	60 (60–65)	62.5 (60–65.75)	0.456
Pre-donation echocardiogram, n (%)			
Dysfunction	7 (10.9)	1 (8.3)	1
Left ventricular hypertrophy	5 (8.6)	1 (10)	1
Ischemia time (min)	146.5 (120–180)	118.5 (102.75–152)	0.106
Extracorporeal circulation time (min)	103 (91.5–118)	96 (84.75–107)	0.185

Values are represented as median (IQR) or n (%). BMI, body mass index; COPD, chronic obstructive pulmonary disease; DBD, donation after brain death; DCD, donation after circulatory death; ECMO, extracorporeal membrane oxygenation; GFR, glomerular filtration rate; PVR, pulmonary vascular resistance.

**Table 2:** Mortality and secondary outcomes of DCD compared to DBD CT recipients.

	DBD n = 64	DCD n = 12	*p*-Value
Morbidity			
Primary graft disfunction	6 (9.4)	0	0.581
Postoperative atrial fibrillation	12 (18.8)	1 (8.3)	0.679
Acute renal failure	36 (56.2)	7 (58.3)	1
Renal replacement therapy in critical care unit	18 (28.1)	7 (58.3)	0.052
Intubation time, hours	9.5	8.5	0.562
Intensive care unit stay, days	7 (5–11.25)	10 (6.75–14.75)	0.142
Total hospital stay, days	21 (17–37.25)	46 (22–70)	0.021
Mortality			
30 d survival	95	100	NA
6-month survival	94	100	NA
1-year survival	93.7	100	NA

Values are represented as median (IQR) or n (%). BMI, body mass index; COPD, chronic obstructive pulmonary disease; DBD, donation after brain death; DCD, donation after circulatory death; ECMO, extracorporeal membrane oxygenation; GFR, glomerular filtration rate; PVR, pulmonary vascular resistance.

**Table 3:** Modified Maastricht classification for DCD [19].

Maastricht Classification	Presentation of Death	Definition
I	Dead in the out-of-hospital setting	1A. Cardiocirculatory death outside hospital with no witness. Totally uncontrolled. 1B. Cardiocirculatory death outside hospital with witnesses and rapid resuscitation attempt. Uncontrolled.
II	Unsuccessful resuscitation	2A. Unexpected cardiocirculatory death in ICU. Uncontrolled. 2B. Unexpected cardiocirculatory death in hospital (ER or ward), with witnesses and rapid resuscitation attempt. Uncontrolled.
III	Awaiting cardiac arrest	3A. Expected cardiocirculatory death in ICU. Controlled. 3B. Expected cardiocirculatory death in OR (withdrawal phase > 30 min). Controlled. 3C. Expected cardiocirculatory death in OR (withdrawal phase < 30 min). (Highly) controlled.
IV	Cardiac arrest while brain death	4A. Unexpected cardio circulatory arrest in a brain-dead donor (in ICU). Uncontrolled. 4B. Expected cardiocirculatory arrest in a brain-dead donor (in OR or ICU). (Highly) controlled.
V	Euthanasia	5A. Medically assisted cardiocirculatory death in ICU or ward. Controlled. 5B. Medically assisted cardiocirculatory death in OR. Highly controlled.

**Table 4:** The largest published clinical studies with DCD hearts.

Study (Author/Country/Year)	Design	Procurement Technique (n)	Donors		Recipients		Outcomes											
			Age (y)	Male (%)	Age (y)	Male (%)	Severe PGD (%)			30-Day Survival (%)			6-Month Survival (%)			1-Year Survival (%)		
							Total	DPP-ESM	TANRP	Total	DPP-ESM	TANRP	Total	DPP-ESM	TANRP	Total	DPP-ESM	TANRP
Messer et al. UK 2020 [7]	CS, SC, R, PSM	DP-ESMP (57) + TANRP-ESMP (22)	35	84	55	77	34	37	26	97	95	100	NA	NA	NA	91	86	100
Kwon et al. USA 2022 [6]	RS, MC, R, PSM	DP-ESMP (175) + TANRP (47)	29	87.3	57	76.4	14.4	NA	NA	99.1	98.8	100	93.5	92.9	96.6	92.5	91.7	96.6
Joshi et al. Australia 2023 [8]	SC, R	DP-ESMP (74)	32	83.8	53	83.8	16	16	-	NA	-	-	NA	-	-	94	94	-
Louca et al. UK, Spain USA, Belgium 2023 [5]	CS, MC, R	TANRP -ESMP (21) + TANRP-SCS (136)	32	83.4	56	78.3	12.8	-	-	96.8	NA	96.8	NA	NA	NA	93.2	NA	93.2
Schroder et al. USA 2023 [11]	CT, MC	DP-ESMP (80)	29.3	93	51.3	73	15	15	-	NA	-	-	94	94	-	NA	-	-
Siddiqi et al. USA 2023 [9]	CS, SC, R	DP-ESMP (21) + TANRP-SCS (101)	26	68	59	84	5.7	NA	NA	96.7	NA	NA	94.3	NA	NA	94.3	NA	NA
Hess et al. USA 2023 [10]	RS, MS, R	DP-ESMP (344) + TANRP (189)	29	86.7	57	79.7	NA	NA	NA	96.8	96.8	98.2	NA	NA	NA	92.8	91.7	93.6

RS, registry study. CS, cohort study. CT, clinical trial. SC, single-center. MC, multicenter. R, retrospective. P, prospective, PSM, propensity score matching. TANRP, thoraco-abdominal normothermic reperfusion. DPP, direct procurement and preservation. ESMP, ex situ machine perfusion. SCS, static cold storage. UK, United Kingdom.

## Data Availability

Upon request.

## Abbreviations

CT	cardiac transplantation
DBD	donation after brain death
DCD	donation after circulatory death
DP	direct procurement
ECMO	extracorporeal membrane oxygenation
ESMP	ex situ machine perfusion
ICU	intensive care unit
KM	Kaplan–Meier
PGD	primary graft dysfunction
RRT	renal replacement therapy
SAT	supra-aortic trunks
SCS	static cold storage
TANRP	thoraco-abdominal normothermic regional perfusion
TCD	transcranial Doppler
WLST	withdrawal of life support treatment
